# Acoustic Emission for Determining Early Age Concrete Damage as an Important Indicator of Concrete Quality/Condition before Loading

**DOI:** 10.3390/ma13163523

**Published:** 2020-08-10

**Authors:** Wiesław Trąmpczyński, Barbara Goszczyńska, Magdalena Bacharz

**Affiliations:** Faculty of Civil Engineering and Architecture, Kielce University of Technology, Al. Tysiąclecia Państwa Polskiego 7, 25-314 Kielce, Poland; bgoszczynska@tu.kielce.pl (B.G.); mbacharz@tu.kielce.pl (M.B.)

**Keywords:** early age concrete, acoustic emission method, damage processes detection before loading, strength of structures

## Abstract

Phenomena occurring during the curing of concrete can decrease its mechanical properties, specifically strength, and serviceability, even before it is placed. This is due to excessive stresses caused by temperature gradients, moisture changes, and chemical processes arising during the concreting and in hardened concrete. At stress concentration sites, microcracks form in the interfacial transition zones (ITZ) in the early phase and propagate deeper into the cement paste or to the surface of the element. Microcracks can contribute to the development of larger cracks, reduce the durability of structures, limit their serviceability, and, in rare cases, lead to their failure. It is thus important to search for a tool that allows objective assessment of damage initiation and development in concrete. Objectivity of the assessment lies in it being independent of the constituents and additives used in the concrete or of external influences. The acoustic emission-based method presented in this paper allows damage detection and identification in the early age concrete (before loading) for different concrete compositions, curing conditions, temperature variations, and in reinforced concrete. As such, this method is an objective and effective tool for damage processes detection.

## 1. Introduction

All engineering projects encounter a range of challenges associated with the most widely used building material, concrete. Being a major problem in current concrete construction, concrete cracking or damage requires a continuous search for new methods and improvement of the existing concrete assessment techniques. This is especially important for fresh concrete, which affects the behavior of concrete under load.

Due to the multilevel nature of concrete, with qualitatively distinct mechanisms taking place during the formation of the concrete, the interaction of various parameters must be considered and the ways to study these relationships and effects have to be found to detect damage. Concrete deterioration occurs primarily through technological cracks (microcracks) and different interfacial properties (cracks) formed at various structural levels, which propagate and initiate operational cracks affecting the usability and strength of concrete elements. The composition of the concrete largely affects its properties. Concrete has high compressive strength and is durable. It can be formed into virtually any shape. Weak points of this material include low tensile strength, shrinkage during the hardening process, and susceptibility to external influences, such as moisture [1], temperature, chemical influences, etc. [2,3].

Particularly important for concrete elements is the early period accompanied by a number of phenomena related to cement hydration [4,5,6].

**Figure 1:**
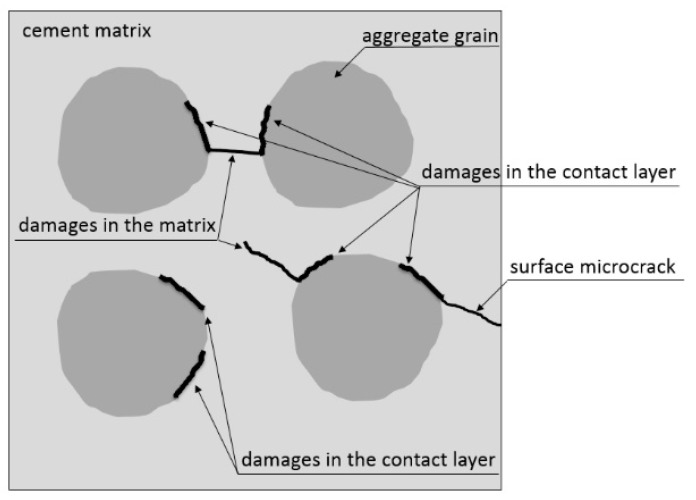
Examples of damages in the concrete microstructure, adapted from [6,7].

Chemical reactions occurring in the cement paste during the hydration process, drying out (water evaporation), the cement paste properties themselves (e.g., bleeding in fresh concrete and temperature changes), as well as volumetric changes due to external factors (temperature and air humidity) cause swelling and chemical, plastic, autogenous, and drying shrinkage. These volume changes of hardening concrete generate natural stress, including “micro” stress. Stresses occur most often in the interfacial transition zones (ITZ) between the grains of aggregate and cement paste and decide on the mechanical properties of these zones and their microcracking. At stress concentrations exceeding the tensile strength of concrete, the microcracks may propagate into the deeper layer of the cement paste or to the surface of the element. Examples of damages in the concrete elements shown in Figure 1.

To mitigate microcracking in concrete, an addition of fly ash, an application of the blast-furnace slag cement or low density aggregate is good practice, as demonstrated in [8]. However, in the case of normal-weight concrete, under the influence of destructive external factors, such as high temperature, frost, and loading, these microcracks can develop into cracks, thereby reducing structural durability and serviceability and in rare cases lead to failures, e.g., walls in tanks [9], concrete slabs [10,11], precast elements [12], or other structural elements [13].

Objective assessment of damage formation and development in concrete, which is independent of the components, additives, and external impacts is essential.

Various non-destructive methods have been used for this purpose [14,15,16]. Acoustic emission (AE) is the technique capable of detecting, classifying [17], and locating [18,19] damage in concrete. Traditional use of acoustic emission methods in the building industry includes the monitoring of damage [20,21] and crack development under load [22,23,24,25,26], cement setting and curing [27,28,29,30,31,32], or an assessment of the ASR (alkali–silica reaction) in concrete [33,34].

The research analyzing stress in concrete is especially related to the acoustic emission phenomenon. The acoustic emission method enables the determination of basic parameters of fracture mechanics necessary to analyze the course of stress affecting concrete destruction. Depending on the grade of concrete tested, the criteria for the estimation of the level of stress were established [35,36]. Another approach aimed at estimating the correlation between acoustic emission and stress in compressed concrete is the technique that relies on the Gutenberg–Richter (GBR) law [37]. It was observed that the event frequency in concrete samples during compression corresponds to about 70% of the maximum stress.

Progressive damage of structural elements (Reinforced Concrete beams—RC beams) under bending is assessed using the Keiser effect by monitoring AE activity during cyclic loading [38]. The Keiser effect is used to estimate the stress to which the structural element was previously exposed. To estimate the Kaiser effect (according to NDIS-2421 by JSNDI—the Japanese Society for Non-Destructive Inspection) two ratios are calculated: the load ratio and calm ratio. Their values are the basis for damage qualification as intermediate, minor, or heavy. Digital image correlation (DIC) techniques supporting the AE method are applied for providing information about the level of damage in the RC beams [39]. The relaxation ratio may also be a good indicator of damage status. During initial stages of loading the deflections increase slightly (loading phase). The element is in a serviceable state up to 50% of the deflection limit. In a higher deflection range (50–85%) the structural element is no longer serviceable. Deflection higher than 85% represents the failure of the element. The acoustic emission method is also used for the observation of the crack mouth opening displacement (CMOD). The different nature of dissipated and emitted energy rates was observed in [38] during the loading process.

The methods performed on concrete subjected to compression and bending do not consider an influence of internal stress on concrete strength. At the initial stage of concrete setting, the cement paste shrinks and meets the resistance of aggregate grains that do not shrink. A self-balancing state of compressive and tensile stress arises. If the tensile stresses in the cement paste exceed the tensile strength, microdefects occur. These defects may form in the matrix and in the interfacial transition zone (ITZ) around the aggregate [4] (first destructive process). Internal microcracks interact with each other; they can join together in a damage network. This happens when the structure surrounding the internal microcracks in the cement paste is not able to transfer accumulated stresses. This is when the second destructive process arises. Furthermore, the heterogeneous increase in temperature in the cross-section of the element, as well as water evaporation from the surface layers causes the stretching in the outer zones and compression of the inner zones of the element. These non-stationary and non-linear temperature and humidity areas generate macrostress in the cross-section [36] that can lead to microcracks on the concrete surface (third destructive process) and then their propagation (fourth destructive process). These destructive processes result in discontinuities in the structure. Local structural defects initiate future destruction of the concrete and may reduce the strength of elements, causing their linear deformation and affecting serviceability functions [4,36,40].

There is no information about the assessment procedure of fresh concrete quality by acoustic emission before loading. In most cases analysis of non-loaded concrete is based on ring-down counting, which involves counting how many times the amplitude passes the fixed threshold or event-counting corresponding to number of AE waves recorded by a single sensor [41]. In these cases, acoustic emission signals the damage (crack formation) without being able to identify the underlying processes. Only some of the AE techniques, such as the methods described in [19,23,24], allow for effective identification and location of the destructive process.

The non-invasive acoustic emission method (modified IADP method—Identification of Active Destructive Processes method) presented in [42,43,44] has been shown to be suitable for investigating defect formation process at the early stage of hardening of young concrete.

The study presented in this paper demonstrates that this method is of a general nature and allows observation and identification of destruction processes regardless of the aggregate used, cement types, admixtures added, hardening conditions, temperature, or the presence of reinforcement. It also enables quantitative assessment of destructive processes, which can be important when assessing the strength properties of concrete.

The method can thus be applied to diagnosing elements made of reinforced concrete, controlling the concrete hardening stage, and supporting decision making (e.g., related to demolding), thereby ensuring the reliability of the structure.

## 2. Materials and Methods

A total of 30 samples (ten concrete series, W2, W3, W4, W5, W6, W7, W8, B2, B3, and B4, of three samples each—A, B, and C) were tested.

Twenty-one samples (W2–W8) were made of C30/37 concrete, six samples (B2, B3) were made of C40/50 concrete and three (W2) of C25/30 concrete. Except for sample W2 (100 mm × 100 mm × 500 mm), all samples had square cross-sections with 150 mm on each side and the length of 600 mm. Samples B2 were made with chemical admixtures (plasticizer and air entraining agent), other samples without admixtures. Samples denoted by “W” were made with limestone aggregate from the Trzuskawica quarry, while these marked with “B” with basalt aggregate from Górażdże quarry. All samples were made with cement CEMI 42,5N—MSR/NA from the Warta cement plant (Cementownia Warta S.A., Trębaczew, Poland) (except B4—CEMIII/A 42,5N—LH/HSR/NA from the cement plant in Małogoszcz, (Cementownia Lafarge Małogoszcz, Poland). The chemical compositions of the cements are compiled in Table 1. Mixture proportions of samples W2–W8 and B2–B4 are listed in Table 2.

Three samples B4 were made with basalt aggregate, blast furnace slag, and cement CEM III without any additions.

**Figure 2:**
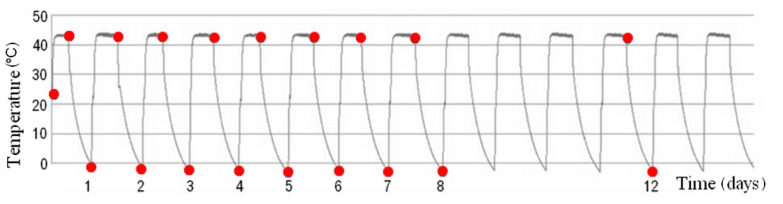
Temperature conditions during the first 14-days of test.

**Figure 3:**
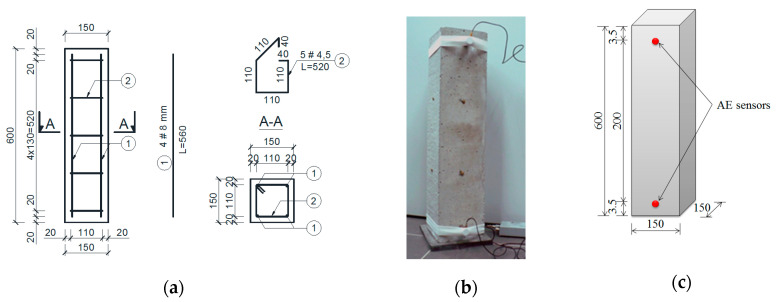
(**a**) Schematic diagram of reinforcement of W4 samples; (**b**) sample during the test; and (**c**) acoustic emission (AE) sensor arrangement (unit: mm).

The W3 and W4 samples after fabrication were cured in water for 10 days and then tested for 58 days under cyclic temperature variations (Figure 2). Additionally, steel reinforcement was embedded in the W4 samples (Figure 3a).

The samples in series W7 were tested for 58 days without water curing at a constant temperature (+22 ± 2 °C).

Before the test, the AE (Acoustic Emission) sensors were attached to one side of each sample (Figure 3b,c).

To provide appropriate conditions, the test stand was developed, comprising of a thermally and acoustically insulated chamber. A list of samples examined is shown in Table 3.

AE signals were recorded for 58 days in 12-h stages.

The proposed identification of active damage processes (IADP) method was presented in [19,21,24] and applied for damage identification and location in reinforced concrete beams under loading [22]. It relies on the study of AE signals produced by the process causing the deterioration of strength properties in structural elements. The results recorded in samples (AE signals) were compared with the reference signals obtained in the laboratory.

Then the modified version of this method was applied to detect damage in young concrete [23,42].

**Figure 4:**
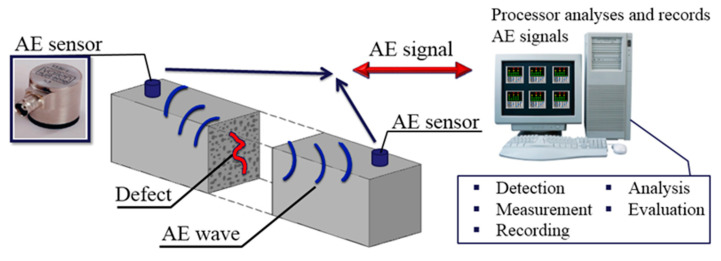
The concept of the method—IADP (Identification of Active Destructive Processes).

The IADP method outline is shown in Figure 4. This concept is based on the comparative analysis of waves generated by defects in concrete (detected by sensors) with a database of reference signals created earlier. Preamplifiers with a gain of 35 dB were used to amplify signals generated by defects. Then the signals were detected, transformed into electric signals, measured, recorded, analyzed, and assigned to the reference signals in the database using Noesis software and unsupervised learning methods.

Figure 3 shows the AE sensors arrangement on the test sample. Two piezoelectric sensors with a gain of 25–80 kHz allow not only detection of destructive processes (AE source) but also finding their linear location.

The preliminary reference signal database was developed based on 12 parameters of the AE signal: counts, counts to the peak, amplitude signal duration, signal rise time, signal amplitude, signal energy, signal strength average, effective voltage, absolute energy, average frequency, reverberation frequency, and initiation frequency. There are four destructive processes, described in [2,3,4,7], which may be a source of AE in freshly made concrete before loading. In [42,43,45] damage processes were ascribed to four signal classes recorded in non-loaded concrete (Table 4).

## 3. Results

### 3.1. Test Results

The method of assessing the quality of early-age concrete must enable identification of internal defects, regardless of the components used for its manufacture or the conditions under which the structure of hardened concrete forms. The potential of the IADP method was analyzed in this context. Damage processes were identified based on the assessment of signal classes recorded during the test.

The AE signals were recorded for 12 h on days: 1–8, 12, 16, 20, 24, 28, 38, 46, and 57 using MISTRAS software. Then the proposed IADP method was used to analyze the signals (hits). The signals from the tests were compared by 12 AE parameters with signals from the database and assigned to particular classes. The reference database was first developed using K-means clustering and then verified in [43].

An analysis was performed on averaged results of the number of recorded signals (hits) with respect to destructive processes assigned to them. Concrete of each series was investigated by 6 sensors (two sensors were attached to one side of each of the three samples (A, B, and C) in a given series). All signals recorded by these 6 sensors capturing the processes were averaged for each series. The number of signals recorded on average by one sensor was analyzed.

The development of damage processes in series W3, W4, W6, and W7 and B2, B3, and B4 is shown in Figures 5, 7, 9, 10, 12, and 13. Corresponding images of side surface cracks (for samples A, B, and C) are presented in Figures 6, 8, and 11, except W6 and B2-B4 samples, because no cracks were observed on their surfaces.

#### 3.1.1. Concrete W3—Curing in Water, Variable Hardening Temperature (+42 to −5 °C)

**Figure 5:**
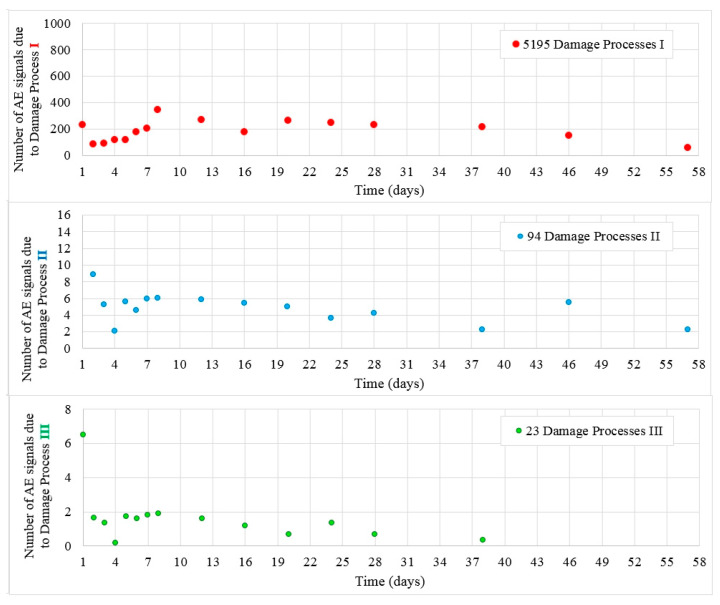
Number of damage processes I–III obtained from W3 samples.

Figure 5 shows the values of destructive processes I–III captured during 56 days in samples W3. Throughout the test, 5195 signals (hits) were assigned to the initiation of internal microcracks (damage process I) and 94 AE signals were assigned to damage process II.

**Figure 6:**
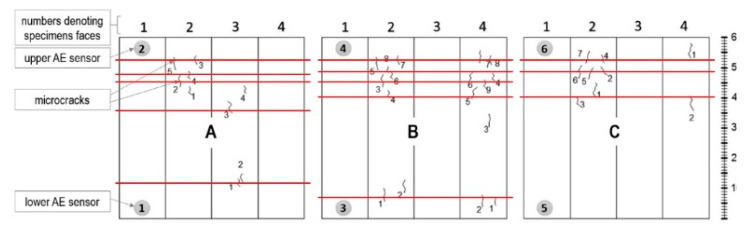
Surface microcrack distribution on sides of the W3 samples. Linear location of destructive processes III (AE signal class 3) in W3 samples obtained by the AE (Acoustic Emission) method is marked in red.

Twenty-three hits assigned to surface microcrack formation (destructive process III) were captured in samples A, B, and C (Figure 6).

#### 3.1.2. Reinforced Concrete W4—Curing in Water, Variable Hardening Temperature (+42 to −5 °C)

**Figure 7:**
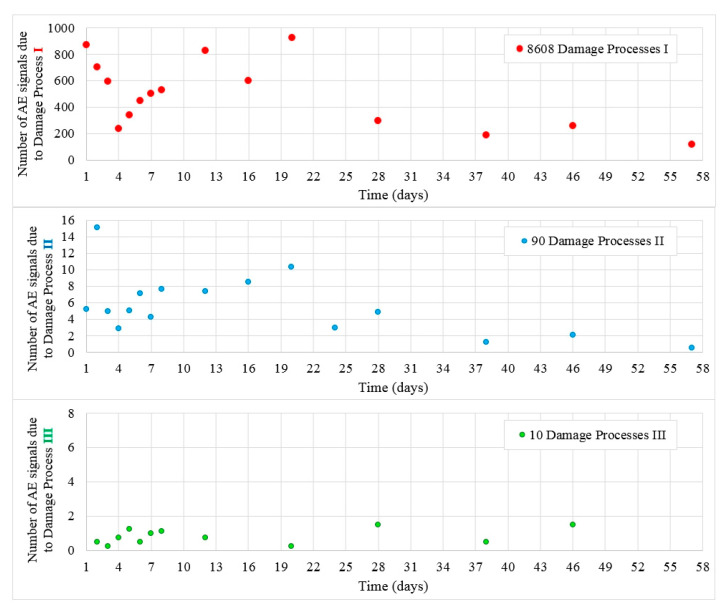
Number of damage processes I–III obtained from W4 samples.

Figure 7 shows the values of destructive processes I–III captured during 56 days in samples W4. Most processes I and II were recorded during 20 days, later their number decreased. Throughout the test, 8608 hits were assigned to the initiation of internal microcracks (damage process I) and 90 AE signals were assigned to damage process II.

**Figure 8:**
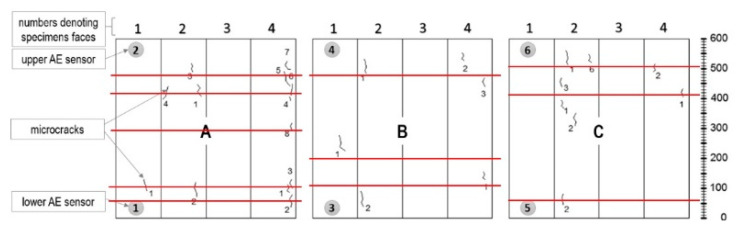
Surface microcracks distribution on sides of the W4 samples. Linear location of destructive processes III (AE signal class 3) in W4 samples obtained by the AE method is marked in red.

Ten signals assigned to destructive process III (surface microcracks formation) were detected in samples A, B, and C (Figure 8) until day 46.

#### 3.1.3. Concrete W6—Curing in Water, Constant Hardening Temperature of 22 °C

**Figure 9:**
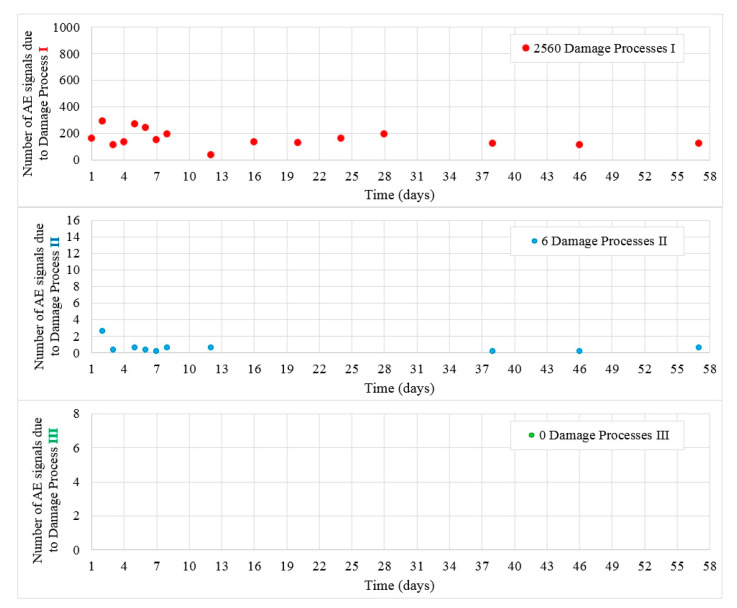
Number of damage processes I–III obtained from W6 samples.

Figure 9 shows the values of destructive processes I and II recorded during 56 days in samples W6. Most processes were recorded during the first week of the test, then their number decreased. Throughout the test, 2560 hits were assigned to the initiation of internal microcracks (damage process I) and 94 AE signals were assigned to damage process II but not on every measuring day.

Class III signals were not captured in the test and no microcracks at the surface of the samples were observed.

#### 3.1.4. Concrete W7—Curing in Water, Constant Hardening Temperature of 22 °C

**Figure 10:**
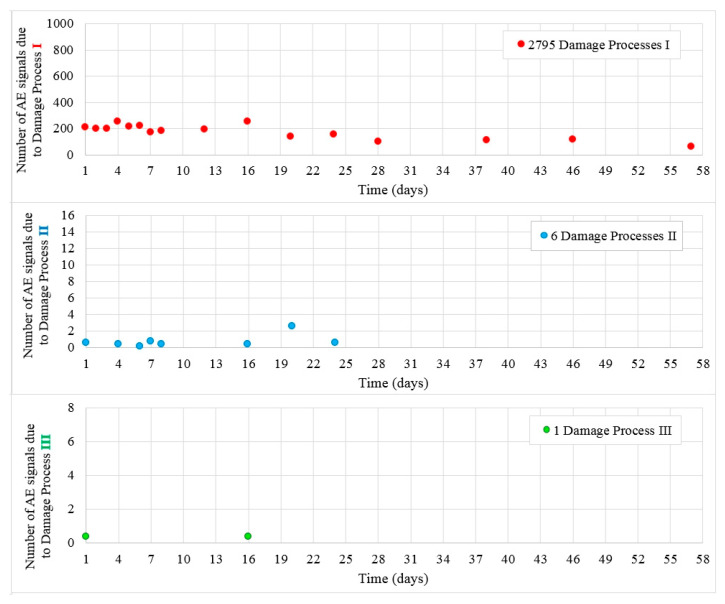
Number of damage processes I–III obtained from W7 samples.

Figure 10 shows the values of destructive processes I-III captured during 56 days in samples W7. Most processes denoted as I were recorded during 20 days, later their number decreased. Throughout the test, 2795 hits were assigned to the initiation of internal microcracks (damage process I) and six AE signals were assigned to damage process II until day 24.

**Figure 11:**
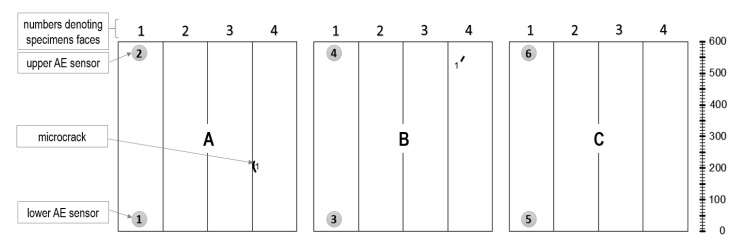
Surface microcracks distribution on sides of the W7 samples.

A few signals Class III assigned to the formation of surface microcracks were recorded in W7 samples. A single surface microcracks were detected on the sides of the samples (Figure 11).

#### 3.1.5. Concrete B2 (with Admixtures)—Curing in Water, Constant Hardening Temperature of 22 °C

**Figure 12:**
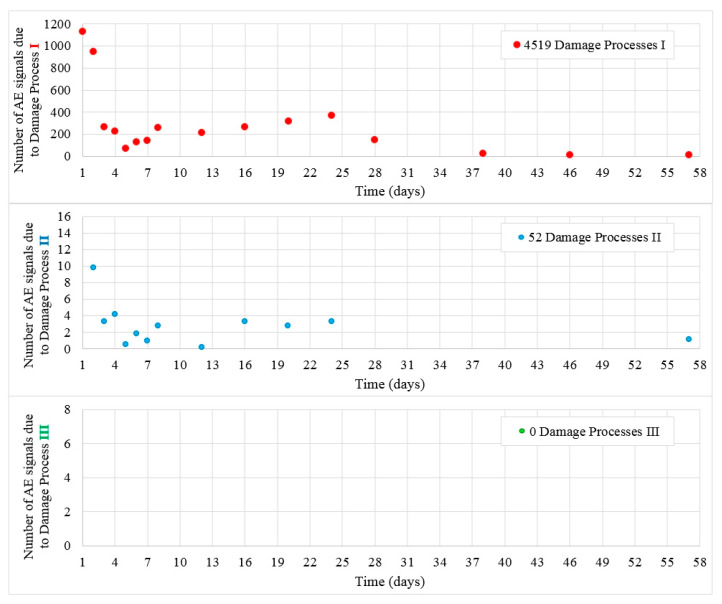
Number of damage processes I–III obtained from B2 samples.

Figure 12 shows the values of destructive processes I and II recorded during 56 days in samples B2. Most processes were recorded during the initial days of the test, then their number decreased. Throughout the test, 4519 hits assigned to the initiation of internal microcracks (damage process I) were recorded together with 52 AE signals assigned to damage process II, which practically faded out after day 24 of the test.

Class III signals were not captured in the test and no microcracks at the surface of the samples were observed.

#### 3.1.6. Concrete B4—Curing in Water, Constant Hardening Temperature of 22 °C

**Figure 13:**
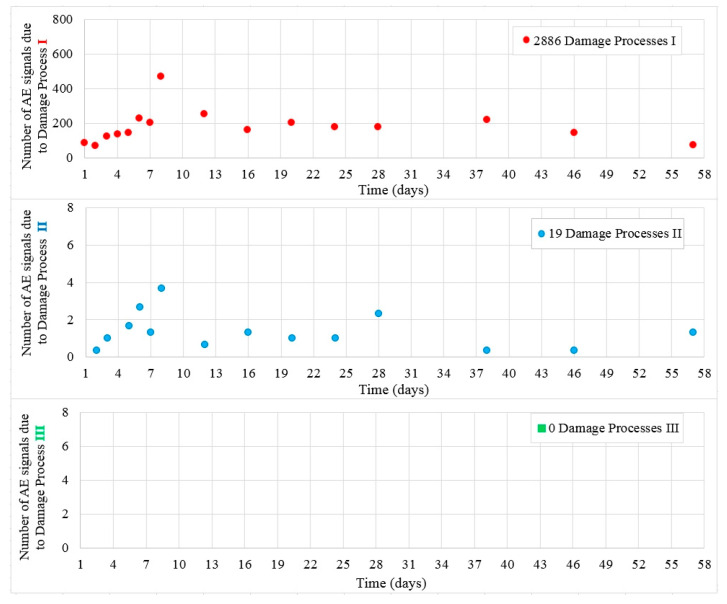
Number of damage processes I–III obtained from B4 samples.

Figure 13 shows the values of damage processes I and II captured during 56 days in samples B4. Throughout the test, 2886 hits assigned to the initiation of internal microcracks (damage process I) and 19 AE signals assigned to damage process II were recorded.

Class III signals were not captured in the test and no microcracks at the surface of the samples were observed.

### 3.2. Destructive Processes Analysis

The analysis of the destructive processes confirmed that the internal structure of the concrete was affected by a range of factors such as aggregate and cement type, curing conditions (especially moisture and ambient temperature) that influence cement hydration, dimensions of the tested element as well as reinforcing bars embedded in the samples.

The identified processes, number of signals and curing conditions are shown in Table 1.

Analysis of the results shows that Class 1 signals were recorded most often in the test. These signals correspond to internal microcrack formation. Most damage processes I were observed during the first week. Their number decreased over time but they did not fade out during 56 days.

The number of Class 2 signals assigned to damage process II (internal microcracks development) was almost an order of magnitude smaller.

Class 3 signals were not recorded in (W2, W6, and B2–B4) concrete samples cured after demolding during 10 days and then hardened at constant temperature (22 ± 2 °C). This indicates that destructive processes III (surface microcrack formation) did not occur, which was confirmed by the observation of the sample.

In addition to tracking the growth of individual destructive processes in time, the number of destructive processes recorded at any given time interval can be also analyzed. Several analyses based on this precise information about damage and damage development in early age concrete were performed.

**Figure 14:**
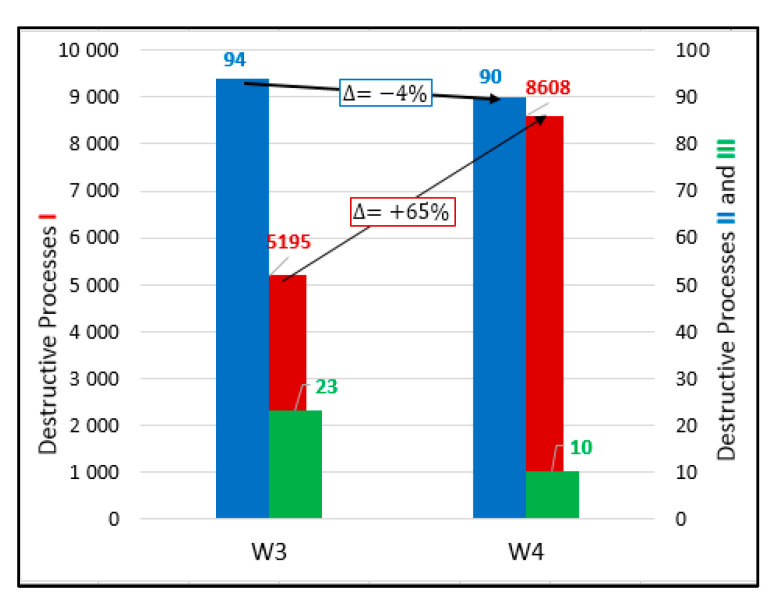
Number of hits accompanying processes I–III recorded in W3 and W4 samples (10 days curing, varied temperature, and with and without reinforcement).

The results selected for W3 and W4 concrete samples (Figure 14) show that in the case when reinforcement was used, the number of damage processes I (internal microcrack formation) increased (in the analyzed concrete samples by about 65%) due to the occurrence of additional interfacial transition zones (ITZ) between reinforcing bars and cement paste. Damage processes II (propagation of internal microcracks) in the analyzed samples slightly decreased and damage processes III (formation of microcracks on the surface of concrete) were limited by embedded reinforcement. 

In Table 5 shown the testing conditions of the samples with the results of number of AE signals and destructive processes in non-loaded concrete obtained by modified IADP acoustic emission method.

**Figure 15:**
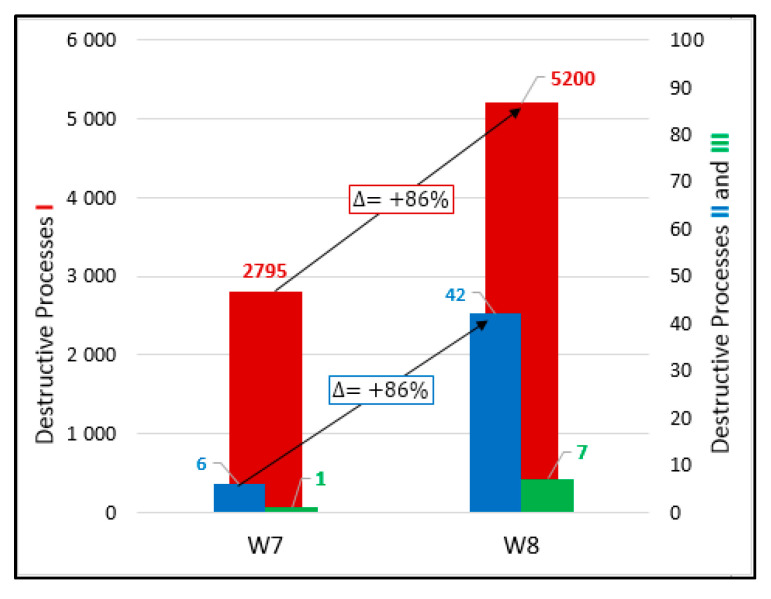
Number of hits accompanying processes I–III recorded in W7 and W8 samples (without curing and constant and varied temperature).

Figure 15 shows an influence of variable temperature on the number of damage processes in concrete hardening without initial curing. In the samples hardened at variable temperatures (−5 to +42 °C), far more destructive processes (I–III) were recorded compared to constant temperature conditions. This indicates a significant impact of the heating and cooling cycles on damage processes development in the early-age concrete, which may influence the strength of hardened concrete.

## 4. Discussion

The presented results for concrete tested under different curing conditions, hardening temperature, aggregate type, concrete strength, dimensions of the sample, presence of the admixtures, and presence of reinforcement show that the proposed AE method is the general method that allows for early age damage identification, damage tracking, and location.

In each of the analyzed cases, it was possible to select damage classes. The emergence of class 1 and class 2 signals does not represent direct effects on the strength level.

Evaluation of concrete strength based on the presented AE method has a qualitative nature. Therefore, it seems expedient to look for a correlation between the intensity of destructive processes and the strength of the concrete obtained.

The data obtained can also be used in several analyses of practical significance such as:

- In the case of hardening at varied temperature, class II damage (internal microcrack development) increases but class III (surface microcracks) decreases in reinforced concrete, which confirms that the reinforcement restricts most dangerous class III damage (Figure 14),
- All damage processes increase in the case of hardening at variable temperature (Figure 15).

## Figures and Tables

**Table 1:** Cement composition (%).

Cement	CaO	MgO	SiO_2_	Al_2_O_3_	Fe_2_O_3_	SO_3_	Na_2_O_eq_	Cl^−^
CEM I	66.03	0.79	21.23	3.66	3.21	2.63	0.43	0.076
CEM III						2.69	0.81	0.066

**Table 2:** Mixture proportions (kg/m^3^).

Symbol	Aggregate 2–16	Sand 0–2	CEMI/CEMIII	Water	Air Entraining Agent	Plasticizer
W3–W8	1110 ^1^	740	338 (CEMI)	169	0	0
W2	1073 ^1^	777	290 (CEMI)	188	0	0
B2	1312 ^2^	691	360 (CEMI)	150	0.36	1.98
B3, B4	1312 ^2^	691	360 (CEMIII)	180	0	0

^1^ limestone aggregate, ^2^ basalt aggregate.

**Table 3:** Sample parameters and testing conditions.

Series	Water Curing (days)	Hardening Temperature	Cement Type	Concrete Class
W2	10	constant 22 ± 2 °C	CEMI	C25/30
W3	10	varied −5 to + 42 °C	CEMI	C30/37
W4 (reinforced)	10	varied −5 to + 42 °C	CEMI	C30/37
W5	10	constant 22 ± 2 °C	CEMI	C30/37
W6 (100 × 100 × 500)	10	constant 22 ± 2 °C	CEMI	C30/37
W7 (without curing)	none	constant 22 ± 2 °C	CEMI	C30/37
W8 (without curing)	none	varied −5 to +42 °C	CEMI	C30/37
B2 (with admixtures)	10	constant 22 ± 2° C	CEMI	C40/50
B3 different agregate type)	10	constant 22 ± 2 °C	CEMI	C40/50
B4	10	constant 22 ± 2 °C	CEM III	C30/37

**Table 4:** Destructive processes by the IADP (Identification of Active Destructive Processes) method.

Denotation	AE (Acoustic Emission) Signal Class	Number of Destructive Process	The Source of the Destructive Process
●	Class 1	I	formation of internal microcracks
●	Class 2	II	propagation of internal microcracks
●	Class 3	III	formation of surface microcracks
●	Class 4	IV	propagation of surface cracks

**Table 5:** Sample parameters, testing conditions, results, and detected damage.

Series	Curing Conditions (Days of Curing)	Hardening Temperature	Cement Type	Concrete Strength after 28 Days (MPa)	Process	No of Signals
W2	10	constant 22 ± 2 °C	CEMI	36.0	I	5980
					II	31
					III	0
W3	10	variable −5 to +42 °C	CEMI	44.5	I	5195
					II	94
					III	23
W4 (reinforced)	10	variable −5 to +42 °C	CEMI	44.5	I	8608
					II	90
					III	10
W5 ^1^	10	constant 22 ± 2 °C	CEMI	40.1	I	2192
					II	9
					III	0
W6 (100 × 100 × 500)	10	constant 22 ± 2 °C	CEMI	40.1	I	2560
					II	6
					III	0
W7 (without curing)	none	constant 22 ± 2°C	CEMI	45.8	I	2795
					II	6
					III	1
W8 ^1^ (without curing)	none	variable −5 to +42°C	CEMI	41.2	I	5200
					II	42
					III	7
B2 ^2^ (with admixtures)	10	constant 22 ± 2 °C	CEMI	63.5	I	4519
					II	52
					III	0
B3 ^2^ different agregate type)	10	constant 22 ± 2 °C	CEMI	55.8	I	2984
					II	18
					III	0
B4	10	constant 22 ± 2 °C	CEM III	48.1	I	2886
					II	19

^1^ The tests and analysis of W5 and W8 samples results were described in [45], ^2^ the test results of the B2 and B3 samples were described in [44].
